# Transformers enable accurate prediction of acute and chronic chemical toxicity in aquatic organisms

**DOI:** 10.1126/sciadv.adk6669

**Published:** 2024-03-06

**Authors:** Mikael Gustavsson, Styrbjörn Käll, Patrik Svedberg, Juan S. Inda-Diaz, Sverker Molander, Jessica Coria, Thomas Backhaus, Erik Kristiansson

**Affiliations:** ^1^ Department of Economics, University of Gothenburg, Gothenburg, Sweden.; ^2^ Department of Mathematical Sciences, Chalmers University of Technology/University of Gothenburg, Gothenburg, Sweden.; ^3^ Department of Biological and Environmental Sciences, University of Gothenburg, Gothenburg, Sweden.; ^4^ Division of Environmental Systems Analysis, Department of Technology Management and Economics, Chalmers University of Technology, Gothenburg, Sweden.

## Abstract

Environmental hazard assessments are reliant on toxicity data that cover multiple organism groups. Generating experimental toxicity data is, however, resource-intensive and time-consuming. Computational methods are fast and cost-efficient alternatives, but the low accuracy and narrow applicability domains have made their adaptation slow. Here, we present a AI-based model for predicting chemical toxicity. The model uses transformers to capture toxicity-specific features directly from the chemical structures and deep neural networks to predict effect concentrations. The model showed high predictive performance for all tested organism groups—algae, aquatic invertebrates and fish—and has, in comparison to commonly used QSAR methods, a larger applicability domain and a considerably lower error. When the model was trained on data with multiple effect concentrations (EC_50_/EC_10_), the performance was further improved. We conclude that deep learning and transformers have the potential to markedly advance computational prediction of chemical toxicity.

## Teaser

Natural language models are successfully used to estimate and improve the accuracy of toxicity modeling from chemical structures.

## INTRODUCTION

Chemical pollution is a driver of biodiversity loss on a planetary scale and a major contributor to the declining ecological status of surface waters across the globe (*1*–*5*). Several adverse environmental effects have been directly associated with chemical pollution, such as the extreme decline in vultures in India (*6*, *7*) and the general decline in bee populations in the Western world (*8*). Chemical pollution also negatively affects humans, with an estimated cost of disease of €157 billion and $340 billion for the European Union and United States, respectively (*9*, *10*). Now, more than 2 million animals are euthanized annually for regulatory purposes (*11*), a number that is expected to increase due to the continuous expansion of the number of chemicals used in society (*12*–*14*).

To ensure that chemicals are used in a safe and sustainable way, increasingly stringent regulatory systems have been implemented over time (*15*, *16*). Environmentally safe concentrations are determined by exposing organisms under controlled conditions to a concentration series of the chemical(s) of interest, determining the concentration at which effects occurs and then applying a safety factor. For the aquatic compartment, data from primary producers, primary consumers, and secondary consumers are typically considered (*17*–*20*).

Computational methods have been suggested as fast and cost-efficient alternatives to experimental data (*21*, *22*). This includes, particularly, quantitative structure-activity relationship (QSAR) methods that use regression or other predictive models (e.g., machine learning–based) to associate differences in chemical structures with changes in toxicological potency. Effects of larger structural alterations are, however, notoriously hard to predict, and most QSAR models are, therefore, often developed using data that are highly stratified, typically based on chemical structure, toxicological effects, species, exposure scenarios, and/or tested end points. This results in narrow applicability domains (ADs), and multiple QSAR models are often required to make predictions for chemicals outside a single class.

Machine learning techniques have been suggested as an approach to integrate large volumes of heterogeneous data into more general models (*23*). For example, deep learning has been used to predict various biological activities, including toxicity, based on chemical structures (*24*–*29*). However, current methods still lack the necessary accuracy and sufficiently large AD required to apply them in regulatory contexts. Existing computational methods have only been able to replace a small proportion of experimental toxicity data (*30*). Thus, improved approaches are needed to ensure that chemical regulation keeps up the pace with the increasing number of chemicals (*12*, *14*).

Recently, transformers, a deep learning methodology originally developed for natural language processing (*31*, *32*), have been shown to be highly efficient at capturing information from biological and chemical structures (*33*–*35*). Transformers use self-attention, a mechanism that infers complex dependencies directly from data to emphasize the parts of the chemical structure that are deemed especially informative. This makes it possible to identify the structural features that are most important for an accurate prediction of chemical toxicity. The rapid accumulation of toxicity data, stemming both from European legislations with a “no data no market” philosophy, and the growing output from the ecotoxicological research community (*36*), has resulted in collections of experimental data for tens of thousands of chemicals, with information on algae, aquatic invertebrates, and fish being especially rich. This paves the way for advanced deep learning methods, such as transformers, to improve the computational predictions of chemical toxicity for these species’ groups.

Here, we describe a transformer-based model that uses chemical structure, together with effect measurements, to predict toxicity. The model shows high predictive performance for aquatic organisms from the three organism groups commonly used in chemical regulation: algae, aquatic invertebrates, and fish. Compared to three commonly used QSAR-based methods, the proposed model has markedly improved accuracy. The transformer-based model also had a larger AD and was, in contrast to the other methods, able to make predictions for all structures in the evaluation dataset. Last, the performance was further improved by combining multiple effect concentrations into a single model. We conclude that transformers markedly advance the computational prediction of chemical toxicity, making it an increasingly attractive alternative to whole-animal toxicity experiments.

## RESULTS

**Fig. 1.**
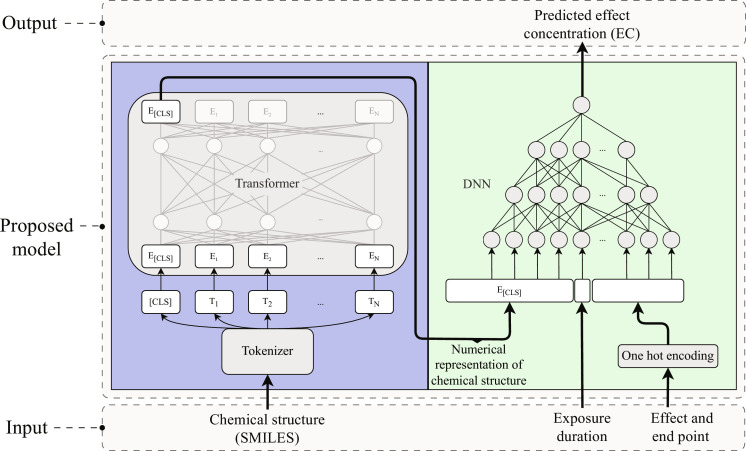
Model architecture. A representation of a molecular structure in the form of a simplified molecular-input line-entry system (SMILES) is first split into tokens (T) and then used as input embeddings (E) for the transformer. The model then uses a pretrained six-encoder layer transformer to interpret the SMILES into a classification embedding (E_[CLS]_) vector of dimension 768, representing the molecular structure with respect to its toxicity (see Materials and Methods). The E_[CLS]_ is then amended with information on exposure duration, effect, and end point and used as input to a DNN. The network then predicts the associated toxicity in the form of an effect concentration (EC_50_ and EC_10_). The parameters and hyperparameters were determined using Bayesian optimization and all weights within the transformer and the DNN were determined during the model training (table S4).

In this study, we present a deep learning model for predicting chemical toxicity based on molecular structure (Fig. 1). The model uses a transformer encoder to derive a numerical representation of the chemical structure, which is then used as input to a deep neural network (DNN) that—together with information on the desired effect, end point, and duration of the exposure—predicts an associated effect concentration (EC_10_ and EC_50_) (*31*, *34*). The transformer and DNN were trained simultaneously, allowing the transformer to up-weight the structural features that are especially important for toxicity. The training data consisted of 147,864 experimentally measured effect concentrations, covering 2321 to 3741 unique chemical structures for three organism groups (Table 1). Stratified sampling was used to reduce the influence of overrepresented chemicals, and Bayesian optimization was used to determine model hyperparameters (*37*). Initially, two individual models were trained for each organism group, one for the prediction of EC_50_ and one for EC_10_ (table S4 and Materials and Methods). The code, data, and trained models are available at https://github.com/StyrbjornKall/TRIDENT). The trained models can be used for inference through the TRIDENT web service available at https://trident.serve.scilifelab.se.

**Table 1.** Overview of the EC_50_ and EC_10_ datasets for fish, aquatic invertebrates, and algae. The datasets were used to train and validate the transformer-based model. The number of unique experimental setups is the number of unique combinations of chemicals, end points, effects, and exposure durations in each dataset. Effect abbreviations: DVP, development; GRO, growth; ITX, intoxication; MOR, mortality; MPH, morphology; POP, population; REP, reproduction.

Dataset	Organism group	End point	Effect	No. of data points	No. of unique chemicals	No. of unique experimental setups	No. of chemicals responsible for more than 50% of data	Concentration (mg/liter) mean (5–95%)*	Exposure duration (hour) mean and SEM*
**Fish EC_50_**	Fish	EC_50_	MOR	52,666	3542	8974	87	27.1 (0.005–155.0)	88 ± 0.63
**Fish EC_10_**	Fish	EC_00_–EC_10_, NOEC	MOR, ITX, DVP, GRO, REP, MPH, POP	19,751	2321	7870	107	16.9 (0.0001–100)	621 ± 10.2
**Aquatic invertebrates EC_50_**	Aquatic invertebrates	EC_50_	MOR, ITX	34,820	3741	9116	98	23.1 (0.0007–140)	78 ± 0.61
**Aquatic invertebrates EC_10_**	Aquatic invertebrates	EC_00_–EC_10_, NOEC	MOR, ITX, DVP, REP, MPH, POP	15,372	2647	6991	118	14.1 (0.0003–100)	311 ± 3.50
**Algae EC_50_**	Algae	EC_50_	POP	13,019	2843	4487	188	25.863 (0.008–144)	91 ± 0.82
**Algae EC_10_**	Algae	EC_00_–EC_10_, NOEC	POP	11,830	2756	4180	184	16.6 (0.003–100)	131 ± 3.48

*These values were calculated from the log_10_-transformed data used to train the models.

**Fig. 2.**
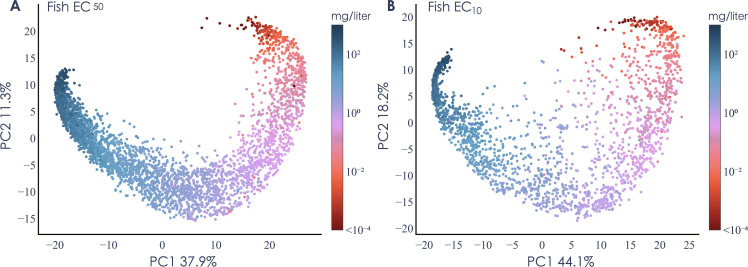
PCA of the numerical presentation of the chemical structures. The plot shows a two-dimensional visualization of the structural representation inferred by the transformer from experimental data on fish for (**A**) EC_50_ (*n* = 3542) and (**B**) EC_10_ (*n* = 2321). The PCA was performed on the 768-dimensional embedding vector (E_[CLS]_) associated with each chemical structure after training was completed. Each dot corresponds to a unique chemical structure, and the distance reflects similarities in their toxicity. The color of each chemical structure is based on the median of the experimentally measured toxicity. The values at the *x* and the *y* axes are the total percent of variation explained by the corresponding principal component.

We first explored how the numerical representation of the chemical structures in the trained model corresponded to toxicity. The transformer derives a 768-dimensional vector from the simplified molecular-input line-entry system (SMILES) that describes the substance toxicity. Principal components analysis (PCA) of the vectors showed that the model organized the chemical structures in a continuous gradient (Fig. 2 and figs. S2 and S3). For example, for fish, the EC_50_ model showed a clear ability to separate toxic and nontoxic chemicals (Fig. 2A), demonstrating that the model properly captures toxicological information from the chemical structure. The EC_10_ models (Fig. 2B and figs. S2B and S3B) followed similar trends but with more chemical structures with deviations, reflecting a slightly higher degree of uncertainty. Analogous patterns could be seen for the other organism groups (figs. S2 and S3).

**Fig. 3.**
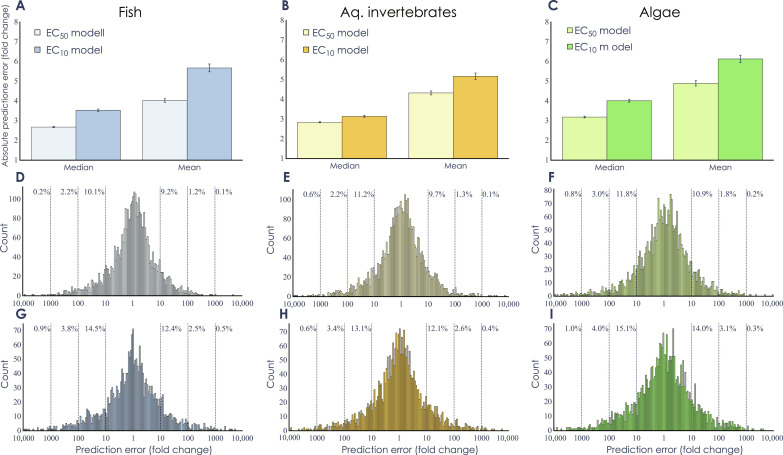
Model performance. (**A** to **C**) Performance as the median and mean absolute prediction error, measured as the absolute fold change (i.e., always using the larger of the measured and predicted value as the numerator when calculating the ratio), determined from 10-fold cross-validations repeated 10 times. In (A), fish EC_50_ model (*n* = 52,666) and fish EC_10_ model (*n* = 19,751), (B) aquatic invertebrate EC_50_ model (*n* = 34,820) and aquatic invertebrate EC_10_ model (*n* = 15,372), and (C) algae EC_50_ model (*n* = 13,019) and algae EC_10_ model (*n* = 11,830). The error bars show the median absolute deviation and the standard error of the mean for the median and mean prediction error, respectively. (**D** to **I**) Histograms of the residual errors (measured as fold change) for the six models: (D) fish EC_50_, (E) aquatic invertebrates EC_50_, (F) algae EC_50_, (G) fish EC_10_, (H) aquatic invertebrates EC_10_, and (I) algae EC_10_. The reported percentage values show the percentage of residuals that are larger than a factor of 10, 100, or 1000, respectively.

The model performance was evaluated using 10-fold cross-validation, where the training and test datasets were created randomly on the basis of unique chemical structures (Table 1 and Materials and Methods). The performance of the model was, thereby, always measured using chemicals that were not included in the training and, thus, new to the model. The highest predictive performances for EC_50_ were seen for fish and aquatic invertebrates, which showed, compared to experimental data, median error factors (the ratio between the prediction and measured data) of 2.66 and 2.82, while the Pearson correlation coefficient (*r*) was 0.69 and 0.73, respectively (Fig. 3, A and B, and table S5). The model performance for algae was slightly lower, with a median error factor of 3.16 and *r* = 0.64 (Fig. 3C and table S5). A closer analysis of the residuals from the fish models showed that close to 80% of all predictions were within a factor of 10 of the experimentally measured data, and only 3.3% of the chemicals had prediction errors larger than a factor of 100 (Fig. 3D). These numbers were similar for aquatic invertebrates (79.1 and 3.5%, respectively; Fig. 3E) and algae (77.3% and 4.8%, respectively; Fig. 3F). The EC_10_ models were able to make predictions with almost the same accuracy (Fig. 3, A to C) where the median errors were a factor of 3.51, 3.12, and 3.99 and the correlation (*r*) is equal to 0.68, 0.74, and 0.60 for the fish, aquatic invertebrates, and algae, respectively (table S5). The corresponding proportion of chemicals deviating more than 10-fold were 73.1, 74.8, and 70.9% (Fig. 3, G to I). For all models, less than 1.5% of the chemical structures had a median error larger than a factor of 1000. As expected, the median error increased with higher structural dissimilarity to the training set (fig. S4).

**Fig. 4.**
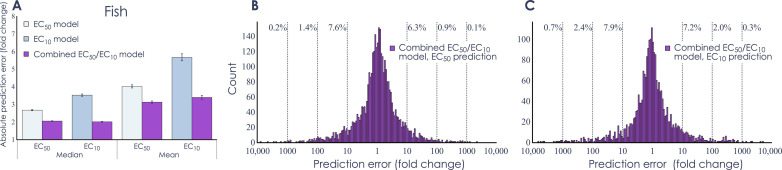
Extended model performance. (**A**) Performance as the absolute median and mean prediction error, measured as the absolute fold change (i.e., always using the larger of the measured and predicted value as the numerator when calculating the ratio) determined from 10-fold cross-validations repeated 10 times. The bars correspond to the fish EC_50_ model (*n* = 52,666), fish EC_10_ model (*n* = 19,751), and the fish extended model for EC_50_/EC_10_ (*n* = 72,417). The error bars show the median absolute deviation and the standard error of the mean for the respective prediction error. (**B** and **C**) Histogram of residual errors (measured as fold change) of the extended fish model when evaluated on the EC_50_ and EC_10_ datasets. The reported percentage values show the percentage of chemicals that are erroneously predicted by a factor of more than 10, 100, or 1000, respectively.

Next, we investigated whether the performance could be further increased by combining predictions of both EC_50_ and EC_10_ in a single extended model. Three extended models were trained (table S4), one for each organism group, and then, as previously, evaluated using 10-fold cross-validation. As we wanted to investigate if data for EC_50_ could improve the prediction of EC_10_, and vice versa, the model was allowed to be evaluated on chemical structure also present in the training data as long as the predicted end point was different. The results showed that the extended models had an increased performance for all three organism groups. For the fish model, the decrease in the median error was 23.3% (from 2.66 to 2.04) and 43.0% (from 3.51 to 2.00) for EC_50_ and EC_10_, respectively (Fig. 4). The increase in performance was similar or even larger for aquatic invertebrates and algae, with decreases in error corresponding to 25.9% (from 2.82 to 2.09) and 37.8% (from 3.12 to 1.94) for aquatic invertebrates EC_50_ and EC_10_, respectively, and 36.7% (from 3.16 to 2.00) and 48.6% (from 3.99 to 2.05) for algae EC_50_ and EC_10_, respectively (fig. S5). The largest performance improvement was seen for chemicals where both EC_50_ and EC_10_ data were available, which had a median error of <2. Similarly, the correlation, when allowing for overlap in structure but not end point, reached 0.80, 0.83, and 0.80 for the fish, aquatic invertebrates, and algae, respectively, thus demonstrating that the extended models can accurately extrapolate to different effect concentrations.

**Fig. 5.**
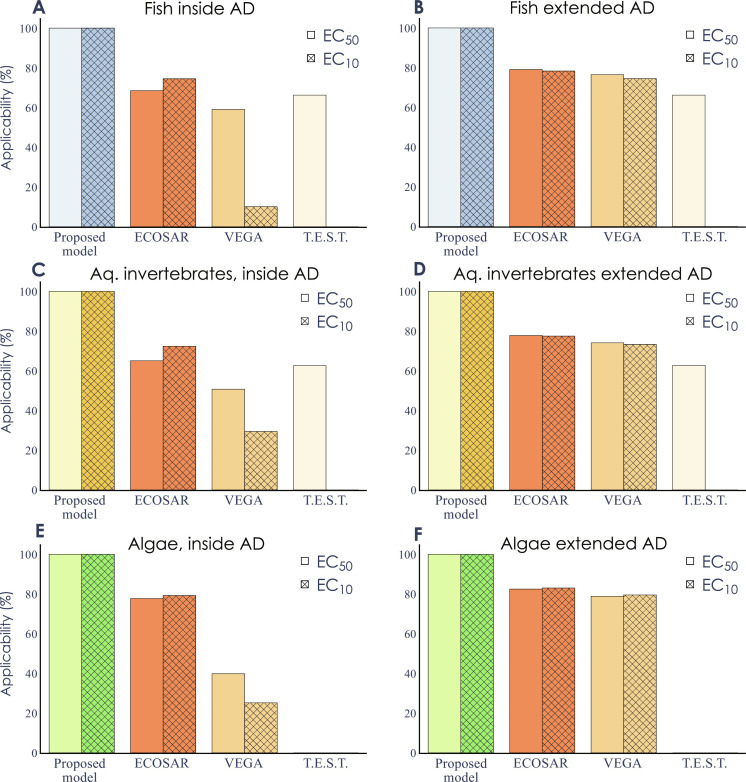
Comparison of ADs. The percentage of chemical structures for which the proposed model, ECOSAR, VEGA, and T.E.S.T. can make predictions. The percentage is reported both for the chemicals that are within the reported AD of the model (**A**, **C**, and **E**) and chemicals that a prediction can be performed for, here called “extended AD” (**B**, **D**, and **F**). The comparisons are based on the total number of unique chemicals included in the full datasets (100%) for [(A) and (B)] fish EC_50_ (*n* = 3542) and fish EC_10_ (*n* = 2321), [(C) and (D)] aquatic invertebrates EC_50_ (*n* = 3741) and aquatic invertebrates EC_10_ (*n* = 2647), and [(E) and (F)] algae EC_50_ (*n* = 2843) and algae EC_10_ (*n* = 2756).

The performance of the transformer-based model was compared to three commonly used QSAR methods for the assessment of chemical toxicity in aquatic organisms (ECOSAR, VEGA, and T.E.S.T.) (*38*–*40*). The transformer-based models were not restricted to specific chemical classes and could, thus, make predictions for all structures included in the training datasets (*n* = 6474 unique structures). In contrast, a large proportion of these chemicals fell outside the ADs of the conventional QSAR methods (Fig. 5). The largest differences in AD were seen for EC_10_ (Fig. 5, A, C, and E), where VEGA was only able to analyze 10 to 30% of the chemical structures (depending on organism group). T.E.S.T. does not have any EC_10_ model and was therefore excluded. Of the three QSAR methods, ECOSAR had the largest AD for EC_10_, and it was able to predict toxicity for more than 75% of the chemical structures. Even when used in the most relaxed settings, under which ECOSAR and VEGA return predictions for chemicals outside of their ADs, these QSAR methods still failed to predict the toxicity of a large proportion of the chemicals (Fig. 5, B, D, and F). Note that there were considerable overlaps between the ADs of ECOSAR, VEGA, and T.E.S.T., resulting in between 19 and 21% (depending on organism group and effect concentration) of the chemicals not being predictable by any of the conventional QSAR methods (fig. S6).

**Fig. 6.**
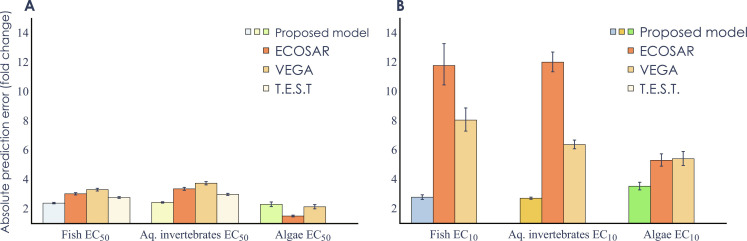
Comparison of performance. (**A** and **B**) Performance as the median absolute prediction error, measured as the absolute fold change between predicted and experimental values (i.e., always using the larger of the measured and predicted value as the numerator when calculating the ratio) for the three QSAR methods such as ECOSAR, VEGA, and T.E.S.T. and from the 10 times repeated 10-fold cross-validations for the transformer-based model. The comparison was done only for chemicals that were within the ADs of all methods but not in the training datasets of any of the methods. In (A), models for EC_50_ (*n* is 734, 752, and 72 for fish, aquatic invertebrates, and algae, respectively). In (B), models for EC_10_ (*n* is 130, 518, and 120 for fish, aquatic invertebrates, and algae, respectively).

Next, we compared the predictive performance of the transformer-based model against the conventional QSAR methods (Fig. 6). To make the comparison fair, we only included chemical structures that were inside the ADs of all methods and that were not included in any of the training datasets used to develop the four compared methods (see Materials and Methods and Supplementary Materials). For the predictions of EC_50_, the transformer-based model had the best accuracy for fish and aquatic invertebrates with a median error corresponding to a factor of 2.35 and 2.40, respectively, while ECOSAR had the best performance for algae with a median error of a factor of 1.41 (Fig. 6A and Table 2). The difference between the conventional methods and the transformer-based model was much larger for the EC_10_ predictions, where the transformer-based model had an error corresponding to a factor of 2.75 and 2.68 for fish and invertebrates, respectively. The second-best method, VEGA, had a predictive error between two and three times larger, corresponding to a factor of 8.02 and 6.35 for fish and aquatic invertebrates, respectively (Fig. 6B and Table 2). For algae, the differences were not as extreme; however, the transformer-based model had an error of a factor of 3.50 compared to 5.27 for ECOSAR, the second-best performing method. In addition, the transformer-based model consistently had a lower error when evaluating the predictive performance individually for each included toxicological effect (figs. S10 and S11).

**Table 2.** Overview of performance comparison. Model performance for the transformer-based model compared to ECOSAR, VEGA, and T.E.S.T. The number of chemicals in the intersect is the number of chemicals that are within the AD of all models but not included in the training datasets. The mean and median absolute error are calculated for this subset of chemicals. The number of chemicals within the extended AD is the number of chemicals for which predictions are provided. This, thus, includes training data for the QSAR methods but excludes training data for the transformer-based model. The number of chemicals within the extended AD does not always match the total number of chemicals included in the training presented in Table 1 as only the data with durations matching the QSAR predictions were included in this comparison. The three rightmost columns report the percentage of chemicals with an error larger than the respective fold difference. Bold indicates best performance.

Dataset	Model	No. of chemicals in the intersect	Mean absolute error	Median absolute error	No. of chemicals (within extended AD)	>1000 [%]	>100 [%]	>10 [%]
Fish EC_50_	Proposed Model	734	**3.19**	**2.35**	3047	**0.3**	**3.2**	**19.0**
	ECOSAR	734	4.44	2.99	1530	3.3	8.6	27.1
	VEGA	734	4.56	3.27	1472	0.4	3.8	23.6
	T.E.S.T.	734	3.82	2.74	1300	2.1	8.2	25.9
Fish EC_10_	Proposed Model	130	**4.57**	**2.75**	2321	**1.4**	**6.4**	**26.8**
	ECOSAR	130	18.1	11.74	1368	9.8	26.2	60.1
	VEGA	130	11.5	8.02	152	3.3	10.5	46.1
	TEST	–	–	–	0	–	–	–
Aquatic invertebrates EC_50_	Proposed Model	752	**3.34**	**2.40**	3110	**0.7**	**3.9**	**20.7**
	ECOSAR	752	5.09	3.33	1758	4.8	10.0	30.7
	VEGA	752	5.29	3.71	1243	1.0	6.3	28.2
	T.E.S.T.	752	3.98	2.95	1548	1.1	5.6	24.9
Aquatic invertebrates EC_10_	Proposed Model	518	**3.97**	**2.68**	2647	**1.1**	**6.0**	**25.2**
	ECOSAR	518	14.34	11.97	1584	5.2	19.1	52.1
	VEGA	518	7.77	6.35	609	1.5	7.6	41.9
	T.E.S.T.	–	–	–	0	–	–	–
Algae EC_50_	Proposed Model	72	4.29	2.59	2325	0.9	4.6	**23.0**
	ECOSAR	72	**1.97**	**1.60**	450	2.7	7.6	24.7
	VEGA	72	2.60	2.23	634	**0.3**	**4.3**	26.3
	T.E.S.T.	–	–	–	0	–	–	–
Algae EC_10_	Proposed Model	120	**6.04**	**3.50**	2756	1.2	7.1	**29.1**
	ECOSAR	120	7.00	5.27	762	3.7	11.4	35.7
	VEGA	120	7.01	5.37	439	**0.2**	**5.5**	30.3
	T.E.S.T.	–	–	–	0	–	–	–

**Fig. 7.**
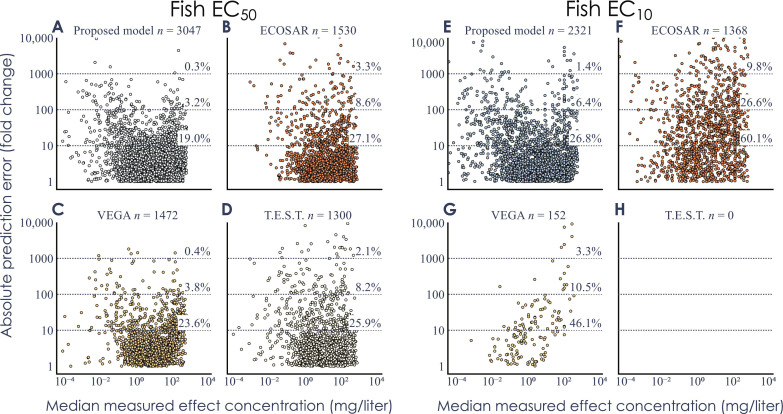
Distribution of the prediction errors. The absolute prediction error, measured as the absolute fold change (i.e., always using the larger of the measured and predicted value as the numerator when calculating the ratio) against the median measured effect concentration for fish for all chemicals within the respective AD of the transformer-based model, ECOSAR, VEGA, and T.E.S.T. (**A** to **D**) Results for EC_50_ predictions. (**E** to **H**) Results for EC_10_ predictions.

The performance of all methods was also assessed by investigating the prediction errors. In this analysis, all chemicals that were inside the AD of each respective method were included, even those that were part of the training sets of the conventional QSAR methods. The results showed that the transformer-based model had the overall highest performance. For the prediction of EC_50_ for fish, only 10 (of 3047, 0.3%) of the chemicals had an error larger than a factor of 1000 and only 91 (3%) had an error larger than a factor of 100 (Fig. 7A). The corresponding error rates for the conventional QSAR methods ranged between 0.4 and 3.3% and 3.8 and 8.6% for a deviation of a factor of 1000 and 100, respectively (Fig. 7, B to D). For predictions of EC_10_ for fish, the differences were even more pronounced, and deviations of a factor of 1000 were between two and seven times more likely for the traditional QSAR methods when compared to the transformer-based model (Fig. 7, E to H). Similar patterns could be seen for aquatic invertebrates (Table 2 and figs. S8 and S9), while the prediction of EC_50_ and EC_10_ for algae VEGA [with a six times lower AD had a smaller percentage with errors >1000 (0.9 and 1.2% for the transformer based method and 0.3 and 0.2% for VEGA, for EC_50_ and EC_10_, respectively). For the median errors, the performance improvement of the proposed transformer-based model was especially pronounced for predictions of EC_10_, where it was reduced by up to a factor of 7.

Last, we evaluated the results from the transformer-based model against a recent model that predicts chemical toxicity [EC_50_ and no effect concentration (NOEC)] in fish (*41*) based on an ensemble of three different machine learning methods (random forest, gradient boosted trees, and support vector regression). Because this model is not publicly available, the evaluation was done on the same set of chemicals as previously published, which showed that the transformer-based model had an improved performance (e.g., root mean square error of 0.83 and 0.97 for the reported method compared to 0.56 and 0.65 for the transformer-based method for EC_50_ and EC_10_, respectively).

## DISCUSSION

In this study, we show that deep learning markedly improves the computational prediction of chemical toxicity to aquatic organisms. We propose an approach that combines transformer with DNNs to derive a general structure toxicity and effect relationship directly from data. This resulted in models with substantially increased ADs that are able to make predictions for large and more diverse sets of chemicals. In addition, the transformer-based models, including the extended models, had superior performance with a median error corresponding to a factor of 2.00 to 3.50 when compared to experimentally measured effect concentrations.

One major difference between the proposed transformer-based model and the conventional QSAR methods is the numerical representation of the chemical structures. Our model uses self-attention, a mechanism originally used to infer complex dependencies in natural language data (*31*, *32*), that has recently been shown to efficiently associate biochemical properties with molecular structures (*33*). During the model training, the representation of the chemical structure is inferred from data by the transformer where structural features that are deemed especially important for toxicity are up-weighed. In contrast, ECOSAR v2.2 contains 263 individual models covering 111 chemical classes only for fish (*42*). In addition, QSAR methods typically use predefined static structural representations, either using single values (*38*) or multidimensional vectors [e.g., fingerprints or other vector molecular descriptors (*27*, *43*)]. Our results, thus, demonstrate that self-attention constitutes an adaptive data-driven approach for estimating the structure-toxicity relationship that improves the predictive performance beyond existing QSAR methods.

The model was trained on a dataset consisting of 147,864 experimental measurements for 6473 chemical structures with species from three organism groups. The high performance of the final model demonstrates that the currently available data is sufficiently rich to train deep learning models that are at least on par, but often superior, to existing methods for predicting chemical toxicity. This suggests that we have reached a tipping point, where data-driven black box models trained on the accumulated data are able to outcompete white box QSAR approaches. This performance gap is expected to grow as more experimental data become available. We, therefore, argue that the adoption and further refinement of data-driven artificial intelligence (AI)–based methods should be prioritized because they have the potential to substantially advance the reliability and applicability of in silico assessment of chemical toxicity. It should, however, be emphasized that data-driven methods are highly dependent on the availability of large volumes of high-quality data. Large efforts are therefore necessary to aggregate information from multiple independent sources to compile datasets of sufficient size. The dataset used in this study included results both from standardized tests and experiments performed by the scientific community. This effort was, however, hampered by the lack of organized ecotoxicity data. The data and metadata describing chemical toxicity are rarely standardized, often limited, incomplete, or inaccessible. In particular, toxicity data generated to comply with European legislations, for example the Registration, Evaluation, Authorisation and Restriction of Chemicals (REACH), is only available as point estimates (e.g., EC_50_, EC_10,_ and NOECs), while data on experimental setups and uncertainties are missing. Also, results from toxicity experiments presented in scientific papers are generally not submitted to data repositories and need to be manually extracted from the texts. Introducing modern FAIR data-sharing principles in both academy, government, and industry would, thus, greatly facilitate the development of data-driven approaches within ecotoxicology (*44*, *45*).

We conclude that AI-based prediction of chemical toxicity offers new means to replace, reduce, and refine the use of animals for experimental purposes. In addition, they allow rapid prescreening of large and diverse bodies of data and will aid in the development of more sustainable chemicals, as well as facilitate the substitution to more benign ones (*46*). This has the potential both to lower societal cost by replacing expensive tests with cheaper computational alternatives and to reduce the burden of disease and impacts on ecosystem services from chemical pollution. Both issues that are becoming increasingly important as the number of chemicals in society grows and exposure to chemical mixtures becomes increasingly complex. Improved computational methods will be vital to ensure safe use of the ever-increasing number of newly found chemicals.

## MATERIALS AND METHODS

### Model design

The model consists of two modules: a transformer encoder and a DNN (*47*). We used a pretrained RoBERTa transformer (ChemBERTa), which consists of 6 encoders, 12 attention heads, and an embedding vector dimension of 768 (*34*). Tokenization of the SMILES was done using the byte pair encoding tokenizer. Tokens generated by the tokenizer could represent single elements or entire subsections of the SMILES. A beginning-of-string token, denoted the “CLS” (classification) token, was added to all sequences during tokenization. All input sequences were then padded or truncated to a maximum length of 100 tokens. The input to the DNN consisted of the CLS token concatenated with the log_10_-transformed exposure duration (hours) and a one-hot encoding vector indicating the type of toxicity end point and effect. The DNN consisted of multiple fully connected layers with a dropout probability of 0.2 and rectified linear unit activation functions. The output layer consisted of a single node predicting the log_10_ effect concentration. The model was implemented in PyTorch v1.10.2 with ChemBERTa loaded from the Huggingface v4.21.1 transformers library (*48*).

### Toxicity data

Data on the toxicity of chemicals, reported as the concentrations at which specific effects of specific sizes were seen, for fish, aquatic invertebrates, and algae were gathered from three sources: REACH dossiers, the United States Environmental Protection Agency (U.S. EPA) database ECOTOX, and the European Food Safety Authority (EFSA) collection of pesticide registration data (*49*–*51*). Toxicity data in REACH was retrieved in August 2020, while the EFSA “openTox” database and the U.S. EPA ECOTOX database were retrieved in November 2020. Toxicity data from the three datasets were merged, and all species’ names were verified using the R package Taxize v0.9.99. The taxonomic groups for all species were harmonized to the same classification as used by the U.S. EPA whenever possible. All NOEC and effect concentration/lethal concentration values reported between 0 and 10% effect were translated to EC_10_ values. All limit tests (data reported as “greater than” or “less than”) were excluded from the analysis. Last, all reported effect concentrations larger than 500 mg/liter were removed to avoid outliers and ensure sufficiently high data quality. Training and validation were performed using log_10_-transformed test exposure and concentration data, thus ensuring that differences in time and concentrations were always relative. All chemical structures, represented as SMILES, were collected by translating the reported CAS from the original datasets using the chemical identity resolver (R package webchem v1.1.2), collecting the first suggested SMILES. The SMILES were then canonicalized through RDKit v2022.03.5.

For algae, we only considered data from population toxicity assays (EC_50_ and EC_10_). For aquatic invertebrates, we considered assays that measured mortality (EC_50_ and EC_10_), intoxication (the term used by the U.S. EPA for the effect group that includes immobility) (EC_50_ and EC_10_), inhibition of reproduction (EC_10_), development (EC_10_) population growth (EC_10_), and morphological effects (EC_10_). For fish, the two datasets contained data for the same end points as the aquatic invertebrate dataset plus data on the growth of individuals (EC_10_).

### Model training

In total, nine individual models were trained. For each organism group (algae, aquatic invertebrates, and fish), we trained models predicting EC_50_ and EC_10_ as well as a combined model predicting both EC_50_ and EC_10_. Model parameters (learning rate, batch size, number of reinitialized encoders, and number of hidden layers in the DNN) were kept identical across organism groups. Thus, three parameter configurations were used in total (i.e., for prediction of EC_50_, EC_10_, and EC_50_/EC_10_, table S4). Specifically, all model configurations used a batch size of 512, three hidden layers (layer sizes 700, 500, and 300), and a learning rate set to 1.5 × 10^−4^, 5.0 × 10^−4^, or 2.0 × 10^−4^ for the prediction of EC_50_, EC_10_, and EC_50_/EC_10_, respectively. In total, the models contained between 84,490,145 and 84,495,745 trainable parameters. The used ChemBERTa transformer version was pretrained on 10 million SMILES (*34*).

Model training was done using the mean absolute error loss function using the AdamW (*52*) optimizer. The transformer encoder and the DNN were trained simultaneously. The learning rate was set to follow a linear schedule with a warmup phase, with a linear increase from 0 to the defined learning rate during 10% of the training steps and then linearly decreased. Layer-wise learning rate decay was employed on the basis of the assumption that the first encoders capture very general language representations and that the last encoders are more task specific (*53*). During training, gradient norms were clipped to 1.0 to avoid exploding gradients (*54*). All training was performed using NVIDIA A100-SXM4-40GB GPUs. Model hyperparameters were determined through Bayesian optimization (Weights and Biases v0.13.1.) based on fivefold cross-validation using the fish datasets (fig. S1 and tables S1, S2, and S3). Stratified sampling with probabilities inversely proportional to the number of data points for each combination of chemical structure, effect, and end point was used to account for skewness in the data.

### Model performance and benchmarking

The models were trained using 10-fold cross-validation that was repeated 10 times. For the models predicting EC_50_ and EC_10_, datasets were split on the basis of the chemical structures to ensure that there was no overlap between chemicals used for training and validation. For the combined EC_50_/EC_10_, data were instead split on the basis of pairs of effect concentration and chemical structure. The predictive performance was determined by first calculating the mean prediction for each datapoint across the repeated 10-fold cross-validation, after which the median residual for each unique combination of chemical, duration, effect, and end point was determined. The overall model performance was then calculated as the weighted mean over all the combinations. Differences in chemical structure were measured as the cosine similarity between the CLS (classification) tokens. During validation, the structural distance between the validation chemical and the training set was measured as the mean cosine similarity between the validation chemical and all chemicals in the training set.

We compared our model against three commonly used QSAR-based methods: ECOSAR v2.2, VEGA v1.1.5, and T.E.S.T. v5.1.1.0 (*38*, *39*, *55*). For the comparison with ECOSAR EC_50_ predictions, 96-hour fish (mortality), 48-hour invertebrate (mortality and intoxication), and 96-hour algae (population growth) measurements were used. For the comparison with VEGA EC_50_ predictions, 96-hour fish (mortality), 48-hour invertebrate (mortality and intoxication), and 72-hour algae (population) measurements were used. For ECOSAR, the predicted Chronic value (ChV) was also, in accordance with current guidelines, divided by the square root of 2 to estimate a predicted NOEC (*56*). For the comparison with ECOSAR and VEGA NOEC predictions, EC_10_ measurements of all durations and effects were used. For the comparison with T.E.S.T., only 96-hour fish EC_50_ (mortality) and 48-hour invertebrate EC_50_ (mortality, intoxication) were used. For ECOSAR, if more than one value per chemical was reported for EC_50_ and ChV, then the lowest reported respective value was collected [as recommended by the user manual (*38*)]. Furthermore, if more than one value was reported for VEGA, then the prediction was taken as the value with the highest reliability score, i.e., “good,” “moderate,” and “low,” in that respective order.

The AD of each method was evaluated on the basis of two criteria. First, counting all compounds where a prediction/experimental value was reported, even if it was reported as being outside the AD and, secondly, only counting compounds within the AD. For the performance comparison, the set of compounds inside the AD of all three QSARs but excluding experimental/training data was used (see Supplementary methods for details).

Last, the model was compared to a machine learning model able to predict fish EC_50_ mortality and NOEC mortality and sublethal effects (*41*). However, because that model was not publicly available, our model was trained on the data included in (*41*) using 10-fold cross-validation and evaluated on the reported measures of root mean square error and percentage of errors of different magnitudes.

## Supplementary Materials

### This PDF file includes:

Supplementary Methods Supplementary Results Figs. S1 to S11 Tables S1 to S5
