# Supplementary material for: Transformers enable accurate prediction of acute and chronic chemical toxicity in aquatic organisms
Source: Sci Adv. 2024 Mar 6;10(10):eadk6669. doi: 10.1126/sciadv.adk6669 (PMC10917336; doi:10.1126/sciadv.adk6669)
Supplement: Supplementary file 1 — Supplementary Methods Supplementary Results Figs. S1 to S11 Tables S1 to S5 [file sciadv.adk6669_sm.pdf]

Science Advances
AAAS

Supplementary Materials for

Transformers enable accurate prediction of acute and chronic chemical toxicity in aquatic organisms

Mikael Gustavsson *et al.*

Corresponding author: Erik Kristiansson, erik.kristiansson@chalmers.se

Sci. Adv. **10**, eadk6669 (2024)
DOI: 10.1126/sciadv.adk6669

This PDF file includes:

Supplementary Methods
Supplementary Results
Figs. S1 to S11
Tables S1 to S5

Supplementary methods

Determining QSAR applicability domains

T.E.S.T., VEGA and ECOSAR each have individual applicability domains and methods for reporting if a chemical is outside of that domain. T.E.S.T. only provides predictions for chemicals inside of its applicability domain. VEGA provides predictions for chemicals outside of the applicability domain but reports these as ‘low reliability’. ECOSAR reports with one or more ‘flags’ or ‘alerts’ if predictions are less reliable.

The QSAR dataset was constructed according to the following 1) for T.E.S.T., all reported predictions were included in the QSAR dataset. 2) for VEGA, all predictions with ‘low’ reliability were considered as outside of the applicability domain. 3) for ECOSAR all predictions with an associated ‘alert’ were considered as outside of the applicability domain, apart from when only the alerts, ‘AcuteToChronicRatios’ or ‘SaturateSolubility’ were reported as these were considered as less severe. Finally, for ECOSAR, predictions for chemicals reported as ‘SHOULD NOT BE PROFILED’ by ECOSAR v2.0 in batch mode were considered as outside of the applicability domain. (ECOSAR v2.0 was used as batch predictions from ECOSAR v2.2 report predictions without flags or alerts when predictions in single compound mode are reported as ‘SHOULD NOT BE PROFILED’). ECOSAR v2.0 was run using both CAS and SMILES as individual inputs to provide full coverage. This mostly reported metals and metal-containing salts.

Extracting which chemicals the QSARs were trained with

Combinations of chemicals and species groups that the QSARs were trained on were identified as follows. 1) For T.E.S.T. and VEGA all combinations with reported ‘experimental’ data. 2) For ECOSAR v2.2, the training data was retrieved from the file:

‘..\ecosarapplication v2_2\Helpful\Consolidated_SAR_MS.pdf’.

Layer-wise Learning Rate Decay (LLRD)

Initial testing with the fish EC_{50} and EC_{10} datasets showed that layer-wise learning rate decay LLRD improved the fine-tuning stability and that LLRD had a very small influence on model performance. All models presented in the main paper are therefore trained using LLRD.

Hyperparameter and parameter sweep setup

Initial testing showed that a fully trainable ChemBERTa transformer had the highest accuracy and consequently neither the embedding layer nor any of the encoders were frozen during the rest of the model development. Initial testing also showed that the dropout rate did not notably affect the accuracy and it was therefore fixed at 0.2.

The final model hyperparameter/-parameter configurations were determined by Bayesian optimization, training the model on the largest datasets per effect concentration (fish EC_{50} and EC_{10}). The optimization included the number of frozen and/or reinitialized encoders and frozen embedding layers in ChemBERTa, batch size, learning rate, dropout rate, as well as the number of hidden layers and number of neurons per layer for the deep neural network. Due to the high number of possible parameter combinations, the optimization was performed in two individual steps. The separation was performed based on the parameters either displaying a decoupling during initial training or because they had little or no impact on model accuracy during initial testing (Table S2, Table S3).

The resulting model configurations were decided based on overall performance (weighted median and mean error) and inter-model uniformity.

Supplementary results

Selection of ChemBERTa version and loss function

Initially, we explored the available pre-trained versions of ChemBERTa and evaluated our model using two loss functions, mean absolute error (MAE/L1) and mean squared error (MSE). ChemBERTa is available with versions pre-trained using either a Byte-Pair Encoding (BPE) or a SMILES tokenizer and with varying amounts of SMILES included in the pre-training. The largest number of unique SMILES included in the pre-training was 10 and 1 million for the BPE and SMILES tokenizers, respectively. Hyperparameter sweeps using Bayesian optimization were performed for both versions, using either the MAE or MSE loss functions. Thus, the optimizer changed both the loss function and learning rate while minimizing the weighted median loss (Table S1). In total 30 individual models per ChemBERTa version were trained (i.e., testing ~15 different learning rates per version and loss function combination). Training was

performed over 30 epochs, using five-fold cross-validation and the best-performing model was used for the evaluation (Fig. S1). This optimization was performed using the largest individual dataset (fish EC_{50}).

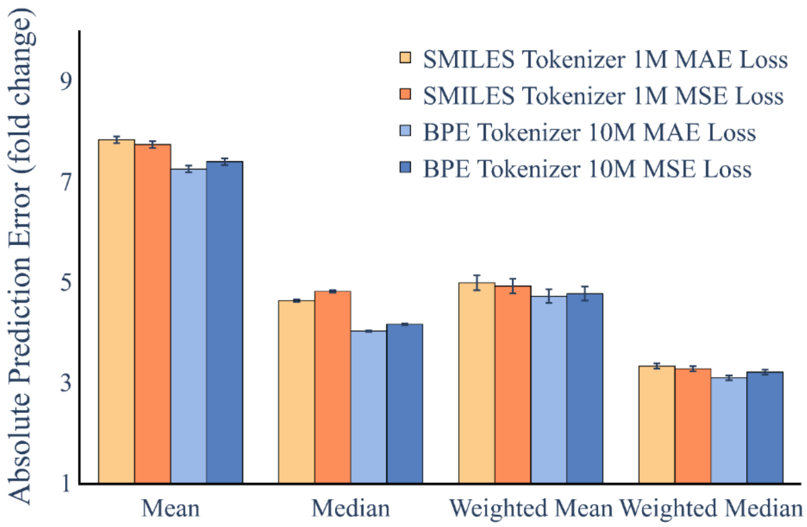

Figure S1: ChemBERTa version and loss-function optimization. Mean, median, weighted mean and weighted median validation loss for the five-fold cross-validation on the fish EC_{50} dataset. Training and validation were performed using a transformer pre-trained either using a byte-pair (BPE) or SMILES tokenizer, and a pre-training-set of either 1 or 10 million SMILES. The error bars show the standard error of the mean when associated with mean errors, and the median absolute deviation (MAD) when associated with median errors. The validation losses were recorded at the epoch where the lowest normalized median validation loss was observed within each fold. The bars are based on the validations from the five 80/20 splits between training and validation. Thus, per fold n = 1934 SMILES was used for the training and n = 483 SMILES were used for the validation.

Principal Component Analysis for invertebrates and algae

PCAs for the CLS-embeddings after the model was trained using the aquatic invertebrate EC_{50} and EC_{10} datasets (Fig. S2) as well as the algae EC_{50} and EC_{10} datasets (Fig. S3).

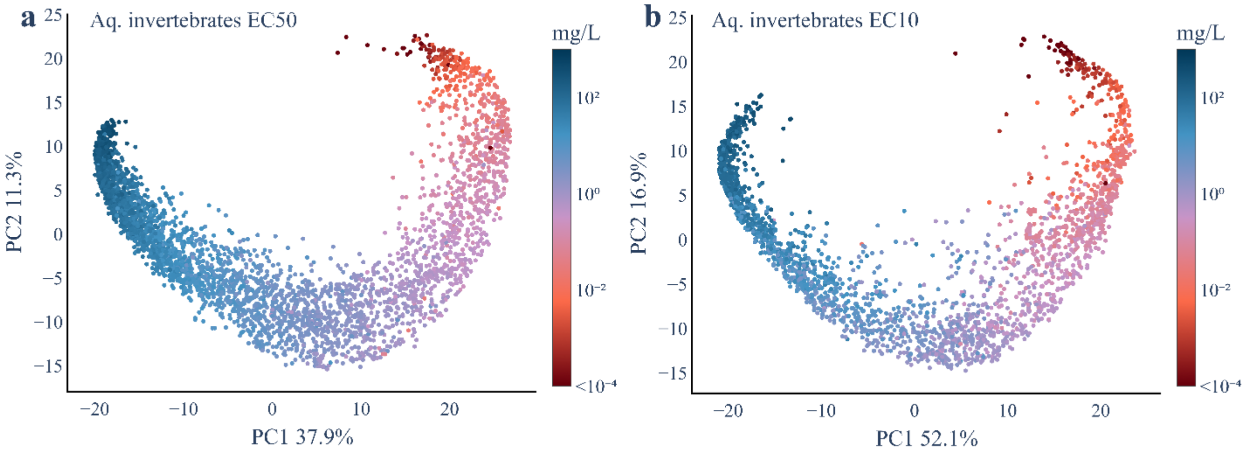

Figure S2: PCA projection of CLS-embeddings from the transformer when trained on aquatic invertebrates EC_{50} and EC_{10} data. Principal Component Analysis of CLS-embeddings from the transformer when trained using the (a) EC_{50} dataset (n = 3741) (b) EC_{10} dataset (n = 2647).

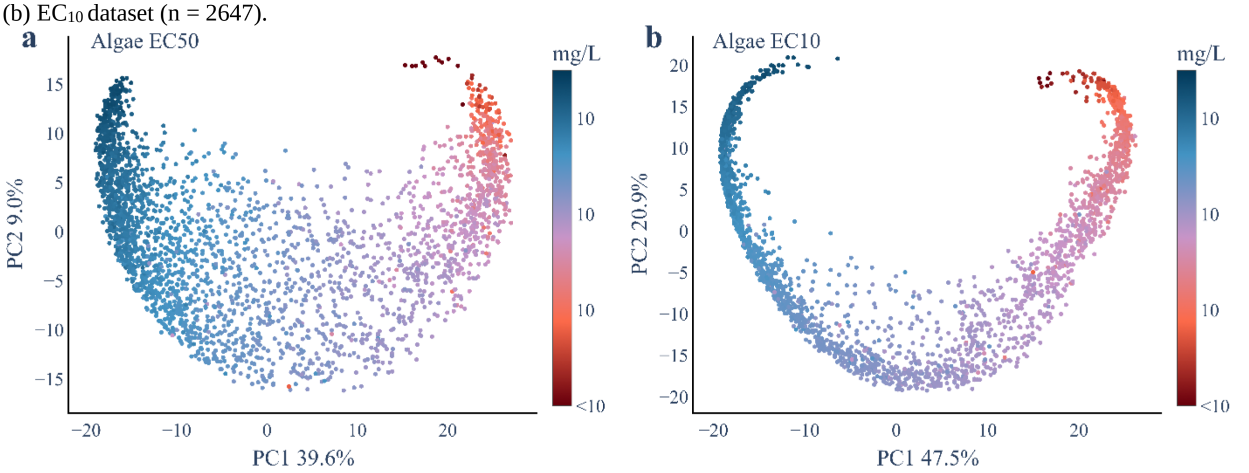

Figure S3: PCA projection of CLS-embeddings from the transformer when trained on algae EC_{50} and EC_{10} data. Principal Component Analysis of CLS-embeddings from the transformer when trained using the (a) EC_{50} dataset (n = 2843) (b) EC_{10} dataset (n = 2756).

Model performance by cosine similarity

The difference in absolute prediction error increased with decreasing between the CLS-embeddings of the validation chemical and the chemicals in the training set (Fig. S4).

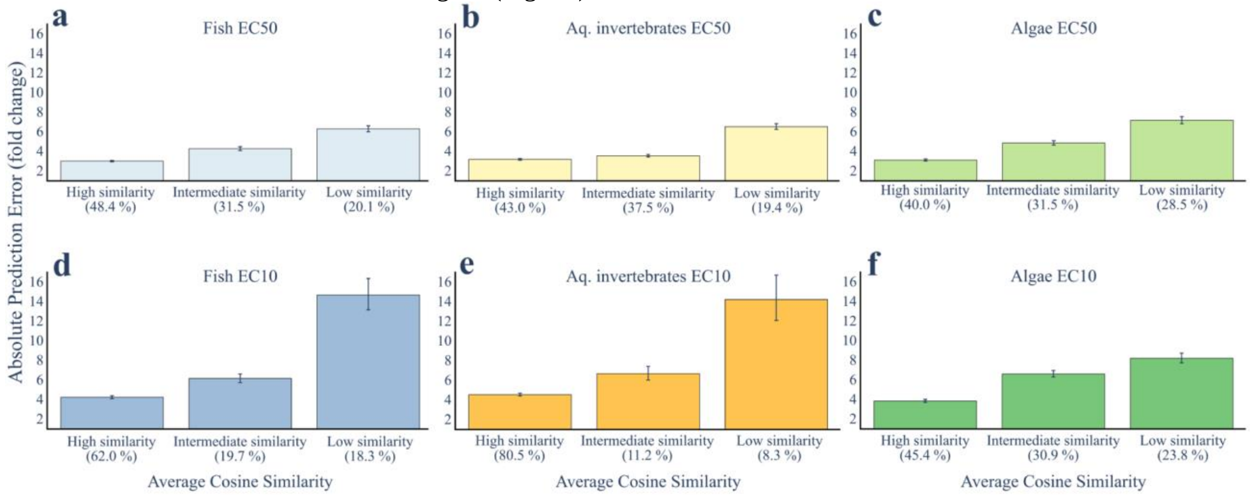

Figure S4: Model performance by cosine similarity. The mean absolute prediction error, measured as the absolute fold change (i.e., always using the larger of the measured and predicted value as the numerator when calculating the ratio), determined from ten-fold cross-validations repeated ten times, split by the median cosine similarity of the validation chemical to the training dataset. High similarity is defined as a cosine similarity > 0.3, intermediate similarity between 0.2 – 0.3 and low similarity < 0.3. In panel (a) fish EC_{50} model (n = 52666), (b) aquatic invertebrate EC_{50} model (n = 34820), (c) algae EC_{50} model (n = 13019), (d) fish EC_{10} model (n = 19751), (e) aquatic invertebrate EC_{10} model (n = 15372), (c) algae EC_{10} model (n = 11830). The error bars show the standard error of the mean. The reported percentage values show the percentage of validation chemicals that belonged to the respective classification during training.

Combined model comparison

The accuracy and residual distribution from the combined EC_{50} and EC_{10} model showed a consistent improvement across all species groups, demonstrating the model's ability to integrate and utilize different types of data (Fig. S5). The improvement is seen both by the Absolute Prediction Error (fold change) and by a decrease in the number of residuals outside a factor of 10, 100 and 1000.

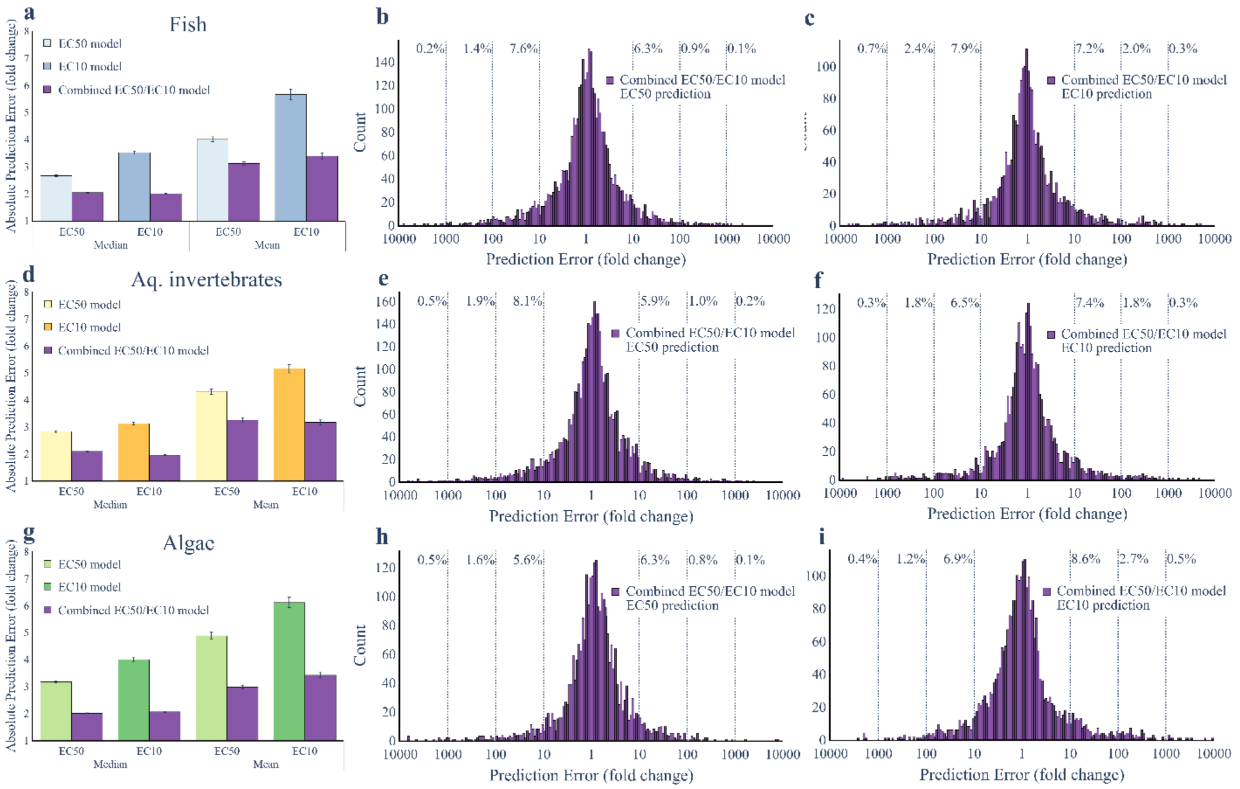

Figure S5: combined model performance fish, aquatic invertebrates, and algae. Panels (a,d,g) show the performance as the absolute median and mean error, measured as the absolute fold-change between predicted and experimental values, determined from the ten-fold cross-validations for the (a) fish EC_{50} model (n = 52666), EC_{10} model (n = 19751), and the model able to predict both EC_{50}/EC_{10} (n = 72417), (d) aquatic invertebrates EC_{50} model (n = 34820), EC_{10} model (n = 15372), and the model able to predict both EC_{50}/EC_{10} (n = 50192), and (g) algae EC_{50} model (n = 13019), EC_{10} model (n = 11830), and the model able to predict both EC_{50}/EC_{10} (n = 24849). The error bars show the median absolute deviation and the standard error of the mean for the respective prediction error. Panels (b-c, e-f, h-i) show the histogram of residuals for the (b-c) fish, (e-f) aquatic invertebrate, and (h-i) algae model able to predict both EC_{50}/EC_{10}, when predictions were evaluated on the EC_{50} and EC_{10} datasets. The reported percentage values show the percentage of chemicals which are erroneously predicted by a factor of more than 10, 100 or 1,000.

Venn diagrams over QSAR applicability

The number of SMILES predictable by each QSAR method, for all chemicals with measured data, as well as all chemicals inside the QSARs applicability domains for the six individual datasets (Fig. S6).

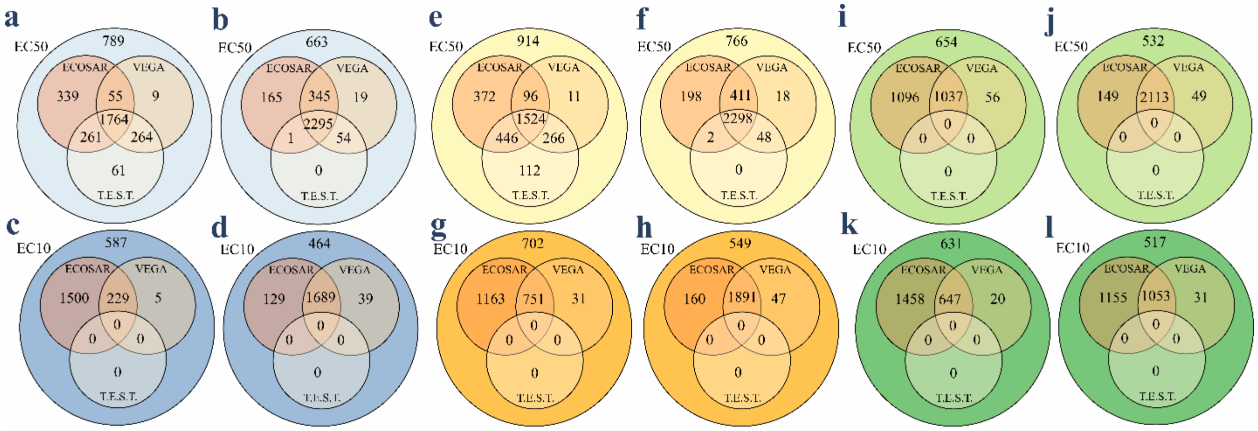

Figure S6: QSAR models applicability intersection. The number of SMILES predictable by each QSAR. (a,b) Number of predictable SMILES both in and outside of the applicability domain for the three QSARs based on all chemicals in the fish EC_{50} dataset. (c,d) The number of predictable SMILES both in and outside of the applicability domain for the three QSARs based on all chemicals in the fish EC_{10} dataset. (e,f) Number of predictable SMILES both in and outside of the applicability domain for the three QSAR tools based on all chemicals in the aquatic invertebrate EC_{50} dataset. (g,h) Number of predictable SMILES both in and outside of the applicability domain for the three QSARs based on all chemicals in the aquatic invertebrate EC_{10} dataset. (i,j) The number of predictable SMILES both in and outside of the applicability domain for the three QSARs based on all chemicals in the algae EC_{50} dataset. (k,l) The number of predictable SMILES both in and outside of the applicability domain for the three QSARs based on all chemicals in the algae EC_{10} dataset.

QSAR accuracy and residual analysis

The accuracies were analyzed for the set of chemicals that were inside the shared applicability domain for all three QSARs (ECOSAR, VEGA, T.E.S.T.) for each of the six datasets, that neither model had been trained on. For our model the validation accuracy from the ten times repeated ten-fold cross-validation is used. Thus, neither model had been trained with the chemicals that are predicted, and no differences in coverage influence the comparison of accuracy. The residuals, per chemical, for all chemicals inside each model's applicability domain was also analyzed and results are summarized in the main text in Table 2. That figure is complemented here for fish (Fig. S7), aquatic invertebrates (Fig. S8) and algae (Fig. S9).

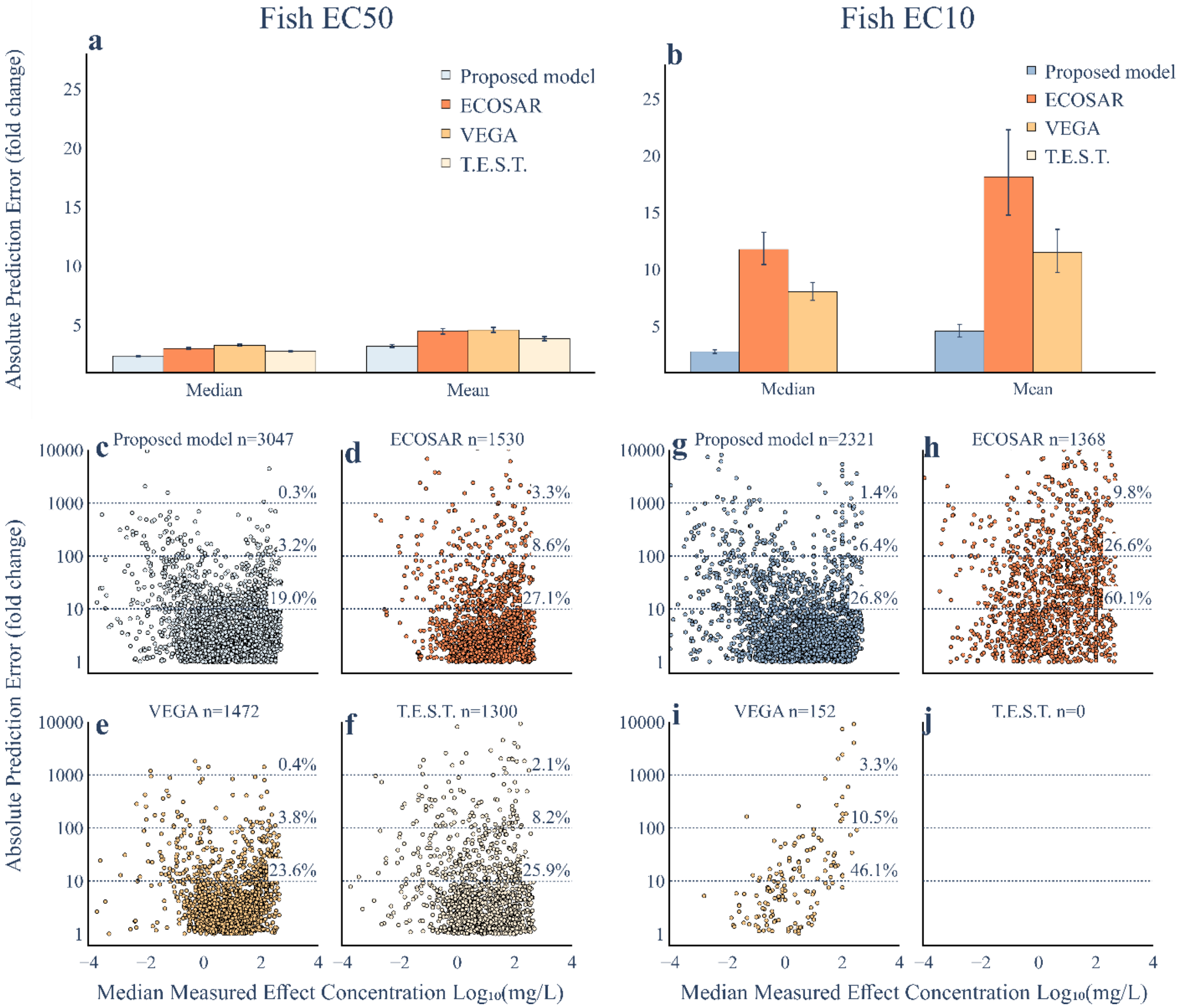

Figure S7: Comparison of model performance and absolute error distribution fish. The mean and median absolute error for the chemicals that are within the applicability domains, but not included in the training, of ECOSAR, VEGA and T.E.S.T. for the models trained using the (a) EC_{50} dataset (n = 734) and (b) EC_{10} dataset (n = 130). (c-j) The absolute error distribution for all chemicals within the applicability domain of the transformer-based model, ECOSAR, VEGA and T.E.S.T.

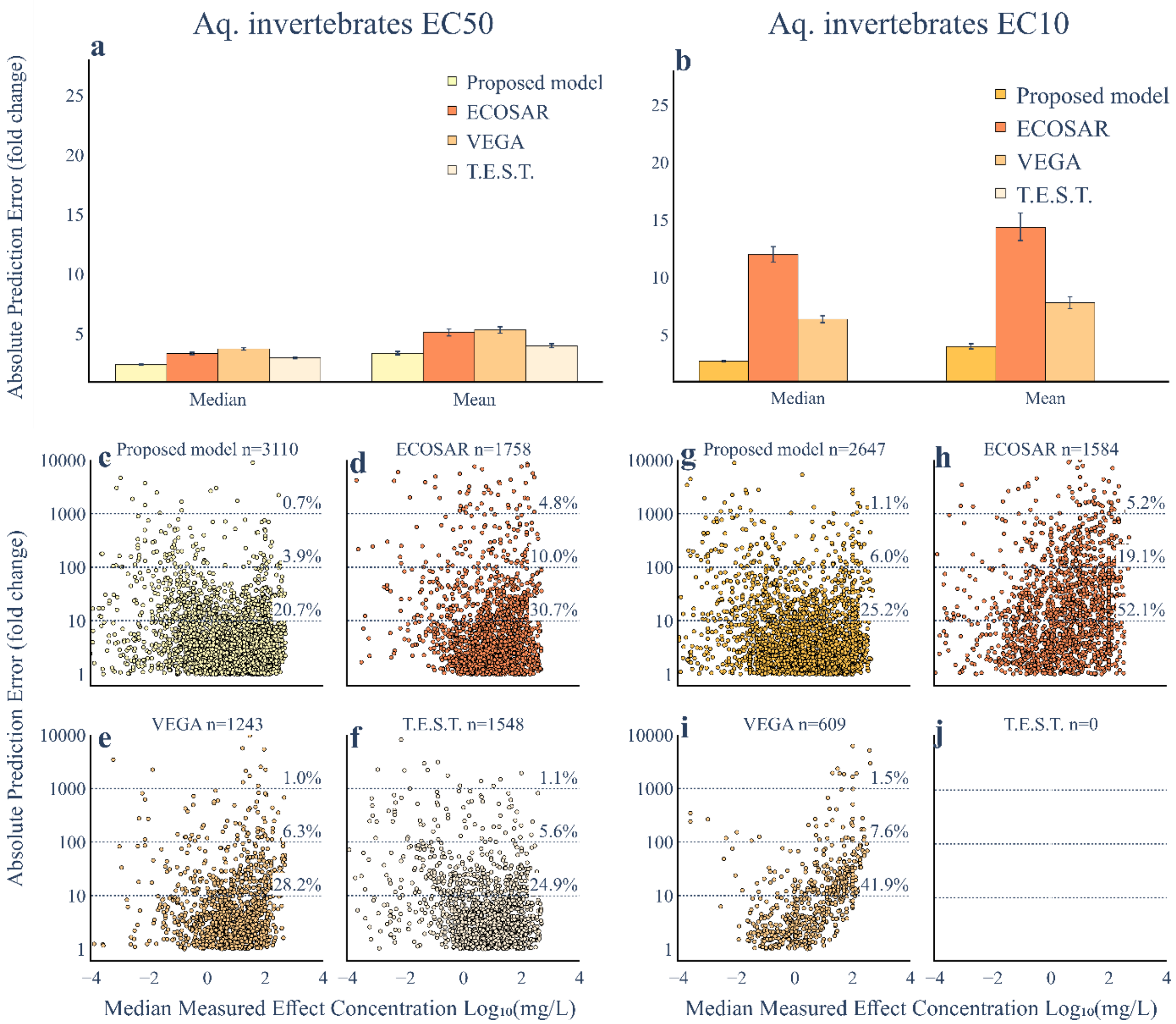

Figure S8: Comparison of model performance and absolute error distribution aquatic invertebrates. The mean and median absolute error for the chemicals that are within the applicability domains, but not included in the training, of ECOSAR, VEGA and T.E.S.T. for the models trained using the (a) EC_{50} dataset (n = 752) and (b) EC_{10} dataset (n = 518). (c-j) The absolute error distribution for all chemicals within the applicability domain of the transformer-based model, ECOSAR, VEGA and T.E.S.T.

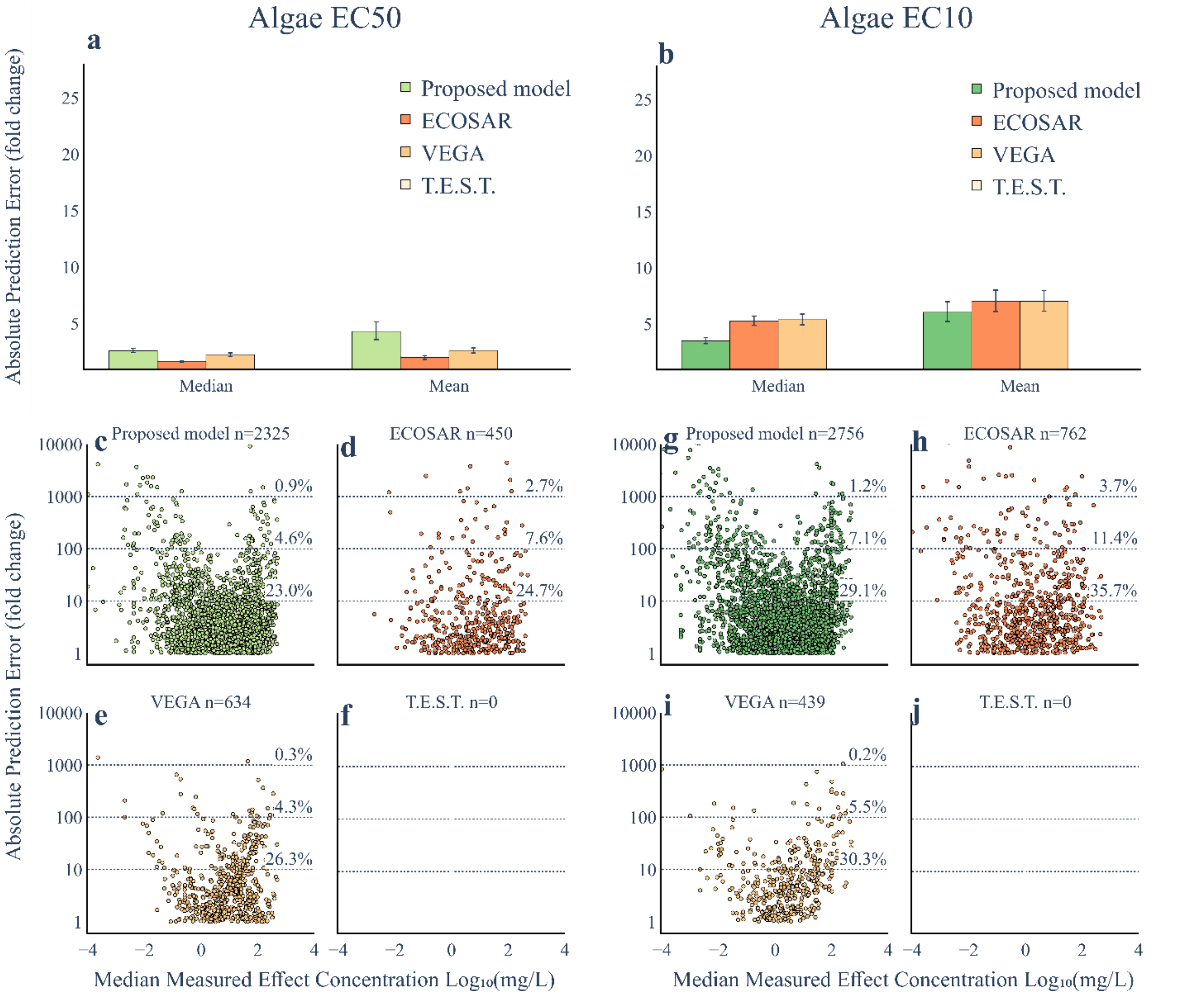

Figure S9: Comparison of model performance and absolute error distribution algae. The mean and median absolute error for the chemicals that are within the applicability domains, but not included in the training, of ECOSAR, VEGA and T.E.S.T. for the models trained using the (a) EC_{50} dataset (n = 72) and (b) EC_{10} dataset (n = 120). (c-j) The absolute error distribution for all chemicals within the applicability domain of the transformer-based model, ECOSAR, VEGA and T.E.S.T.

QSAR residual analysis per effect type for EC10 datasets

The residual error was determined individually for each effect to ensure that the error distributions within the fish and aquatic invertebrate EC_{10} datasets were not dependent on the measured effect (Fig. S10, Fig. S11). The proportions of errors exceeding 10, 100 and 1000 absolute fold prediction errors show that the model performs well also within each effect individually. Note, that algae are not presented as the model only predicts one population effects.

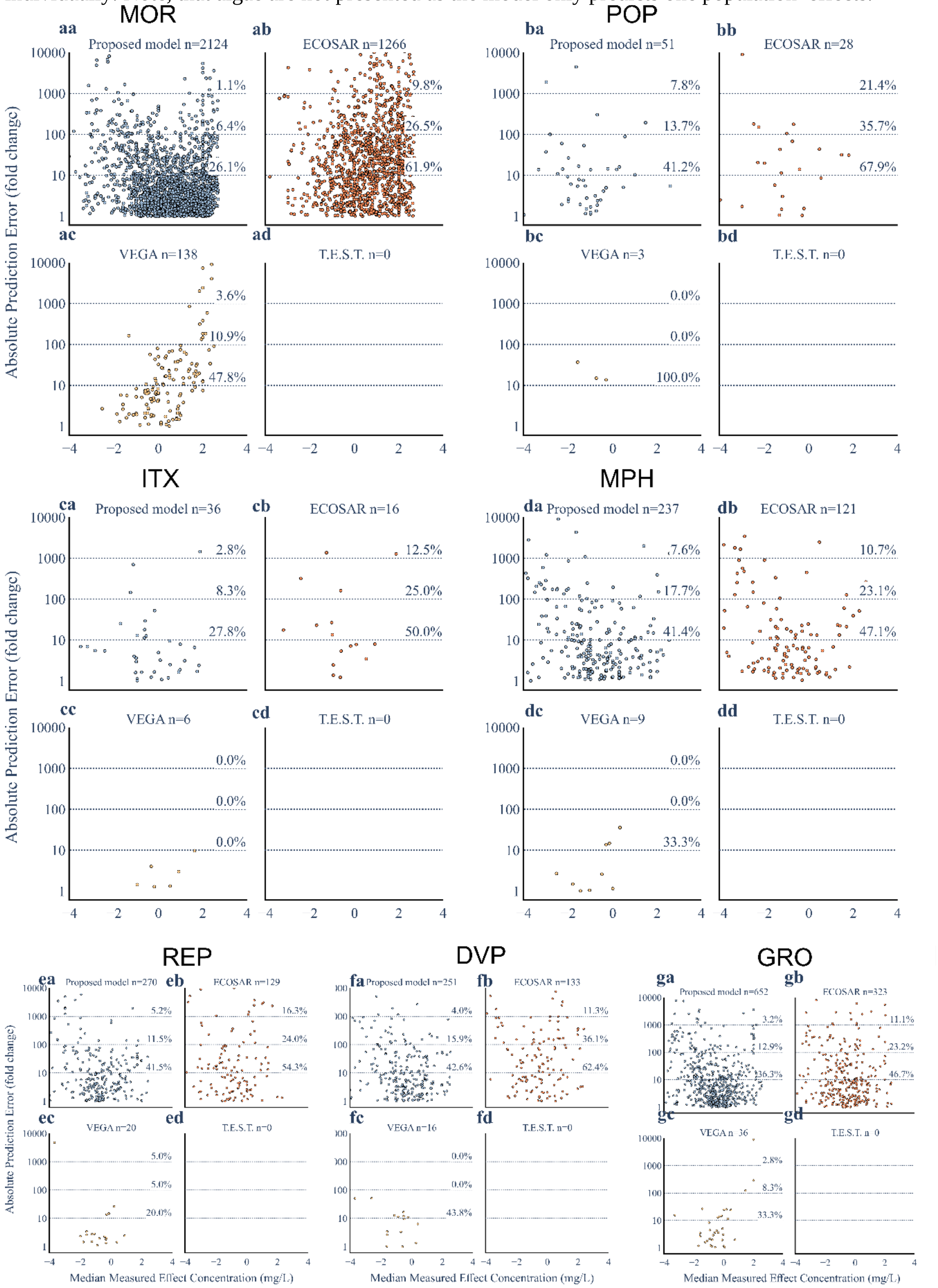

Figure S10: Absolute error distribution per effect fish. The absolute error distribution for all chemicals trained using the fish-EC_{10} dataset for the transformer-based model, ECOSAR, VEGA and T.E.S.T., split by the effects that are inside the applicability domain of our model. *Effect abbreviations: DVP = development, GRO = growth, ITX = intoxication, MOR = mortality, MPH = morphology, POP = population, REP = reproduction.*

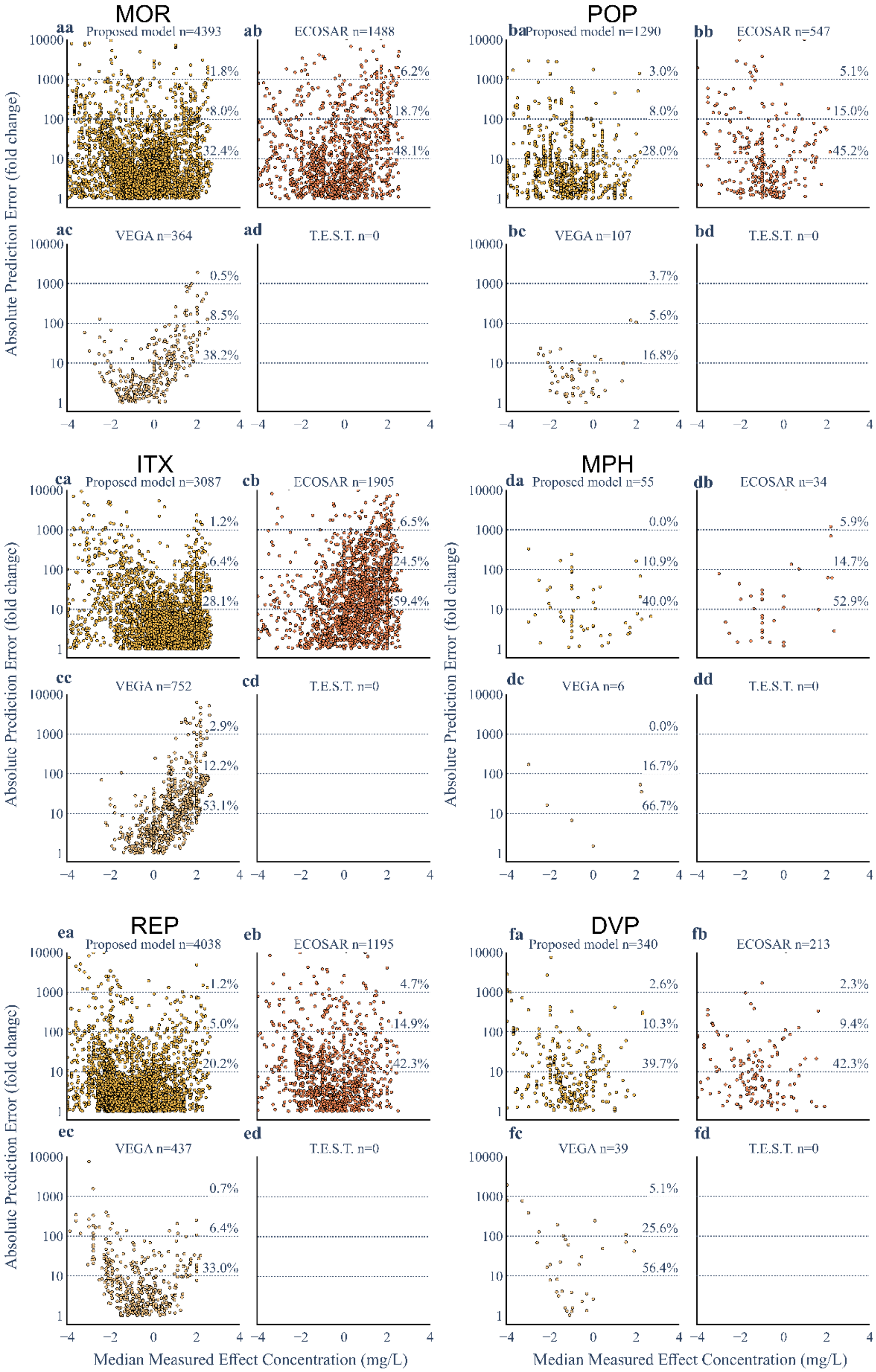

Figure S11: Absolute error distribution per effect invertebrates. The absolute error distribution for all chemicals trained using the aquatic invertebrate EC_{10} dataset for the transformer-based model, ECOSAR, VEGA and T.E.S.T., split by the effects that are inside the applicability domain of our model. *Effect abbreviations: DVP = development, ITX = intoxication, MOR = mortality, MPH = morphology, POP = population, REP = reproduction.*

Table S1: ChemBERTa version and loss-function optimization. Parameter/Hyperparameter sweep to determine the best performing ChemBERTa version and loss function based on the fish EC_{50} dataset. This sweep uses a Bayesian optimizer and a five-fold cross-validation with the average median loss as the target metric. The choice of a log-uniform distribution for the learning rate ensures that learning rates from different magnitudes are equally likely to be tested.

		**EC_{50} model development**	
**Parameter/Hyperparameter**	**Distribution**	**Interval**	**Result**
**Learning rate**	Log-uniform	[1e-3,1e-6]	- [a]
**ChemBERTa version**	Categorical	[SMILES_tokenized_PubChem_shard00_160k, PubChem10M_SMILES_BPE_450k]	PubChem10M_SMILES_BPE_450k
**Loss function**	Categorical	[MAE Loss, MSE Loss]	MAE Loss
**FIXED VALUES**			
Dataset		*Fish EC_{50}*	
Epochs		*30*	
Batch size		*512*	
Dropout		*0.2*	
Number of frozen encoders		*0*	
Frozen embedding layer		*False*	
Number of reinitialized encoders		*0*	
Number of hidden layers		*2*	
Hidden layer sizes		*[350,20]*	

[a] *The resulting learning rate is not specifically of interest, but an interval is necessary as the loss function varied between the tests.*

Table S2: Batch size sweep. Hyperparameter/parameter optimization to determine batch size based on the fish EC_{50} dataset performed using Bayesian optimization with the average median loss across a five-fold cross-validation as the target metric. The choice of a log-uniform distribution for the learning rate ensures that learning rates from different magnitudes are equally likely to be tested.

		**EC_{50} model development**	
**Parameter/Hyperparameter**	**Distribution**	**Interval**	**Result**
**Learning rate**	Log-uniform	[1e-3,1e-6]	- [a]
**Batch size**	Categorical	[512,256,128,64]	512
**FIXED VALUES**			
Dataset	*Fish EC_{50}*		
Epochs	*30*		
Dropout	*0.2*		
Number of frozen encoders	*0*		
Frozen embedding layer	*False*		
Number of reinitialized encoders	*0*		
Number of hidden layers	*2*		
Hidden layer sizes	*[350,20]*		
ChemBERTa version	*PubChem10M_SMILES_BPE_450k*		

[a] The resulting learning rate is not specifically of interest, but an interval is necessary as the batch sizes vary. The learning rate will therefore be subject to change in subsequent sweeps.

Table S3: Learning rate, number of reinitialized encoders, hidden layers and hidden layer size optimization. Hyperparameter/parameter optimization to determine the remaining parameter configurations for all three models. This is performed using Bayesian optimization with the average median loss across a five-fold cross-validation as the target metric. The choice of a log-uniform distribution for the learning rate ensures that learning rates from different magnitudes are equally likely to be tested.

		**EC_{50} model development**		**EC_{10} model development**		**EC_{50}/EC_{10} combined model development**	
**Parameter/Hyperparameter**	**Distribution**	**Interval**	**Result**	**Interval**	**Result**	**Interval**	**Result**
**Learning rate**	Log-uniform	[1e-3,1e-6]	1.5e-4	[1e-3,1e-6]	5e-4	[1e-3,1e-6]	2e-4
**Number of reinitialized encoders**	Categorical	[0,1]	0	[0,1]	0	[0,1]	0
**Number of hidden layers**	Categorical	[2,3,4]	3	[2,3,4]	3	[2,3,4]	3
**First hidden layer size**	Int-uniform	[768,300]	700	[768,300]	700	[768,300]	700
**Subsequent hidden layer size**	Int-uniform	[500,20]	[500,300]	[500,20]	[500,300]	[500,20]	[500,300]
**FIXED VALUES**							
Dataset	*Fish EC_{50}, Fish EC_{10}, and combined Fish EC_{50} & Fish EC_{10}*						
Epochs	*30*						
Batch size	*512*						
Dropout	*0.2*						
Number of frozen encoders	*0*						
Frozen embedding layer	*False*						
ChemBERTa version	*PubChem10M_SMILES_BPE_450k*						

[a] The first hidden layer size is allowed a different interval than subsequent layers. Due to the definition of the sweep, subsequent hidden layers share the same interval, but can assume different sizes. The result should be read as [size of sub. Layer 1, size of sub. Layer2]

Hyperparameter and parameter sweep results

The final model parameter and hyperparameter configurations are presented in Table S4.

Table S4: Final model hyperparameters. The set of hyperparameters that yielded the highest performance for the model. The parameters were set after performing Bayesian optimization with Weights and Biases v0.13.1. The model configurations are used for all species groups and were derived from the largest individual dataset (fish EC_{50}, fish EC_{10} and fish EC_{50}/EC_{10}).

Model	Learning rate	Batch size	Number of reinitialized encoders	Number of hidden layers	Hidden layer sizes
**EC_{50} model**	1.5e-4	512	0	3	[700, 500, 300]
**EC_{10} model**	5e-4	512	0	3	[700, 500, 300]
**Combined EC_{50}/EC_{10} model**	2e-4	512	0	3	[700, 500, 300]

Pearson correlation coefficients

Table S5: Pearson correlation coefficients. The correlation coefficients per dataset. For the extended model the correlation coefficients have been determined when the validation chemicals are allowed to present also in the training dataset, as long as the predicted endpoint was different (overlap).

**Organism Group**	**Model version**	**Pearons correlation coefficeint (r)**
**Fish**	EC50	0.688
**Fish**	EC10	0.680
**Fish**	Combined EC_{50}/EC_{10} (overlap)	0.797
**Aquatic invertebrates**	EC50	0.733
**Aquatic invertebrates**	EC10	0.739
**Aquatic invertebrates**	Combined EC_{50}/EC_{10} (overlap)	0.827
**Algae**	EC50	0.643
**Algae**	EC10	0.595
**Algae**	Combined EC_{50}/EC_{10} (overlap)	0.800
